# 2-D Unitary ESPRIT-Like Direction-of-Arrival (DOA) Estimation for Coherent Signals with a Uniform Rectangular Array

**DOI:** 10.3390/s130404272

**Published:** 2013-03-28

**Authors:** Shiwei Ren, Xiaochuan Ma, Shefeng Yan, Chengpeng Hao

**Affiliations:** The State Key Laboratory of Acoustics, Institute of Acoustics, Chinese Academy of Science, Beijing 100190, China; E-Mails: maxc@mail.ioa.ac.cn (X.M.); sfyan@mail.ioa.ac.cn (S.Y.); haochengp@mail.ioa.ac.cn (C.H.)

**Keywords:** direction of arrival estimation, two-dimensional unitary transformation, coherent signals

## Abstract

A unitary transformation-based algorithm is proposed for two-dimensional (2-D) direction-of-arrival (DOA) estimation of coherent signals. The problem is solved by reorganizing the covariance matrix into a block Hankel one for decorrelation first and then reconstructing a new matrix to facilitate the unitary transformation. By multiplying unitary matrices, eigenvalue decomposition and singular value decomposition are both transformed into real-valued, so that the computational complexity can be reduced significantly. In addition, a fast and computationally attractive realization of the 2-D unitary transformation is given by making a Kronecker product of the 1-D matrices. Compared with the existing 2-D algorithms, our scheme is more efficient in computation and less restrictive on the array geometry. The processing of the received data matrix before unitary transformation combines the estimation of signal parameters via rotational invariance techniques (ESPRIT)-Like method and the forward-backward averaging, which can decorrelate the impinging signals more thoroughly. Simulation results and computational order analysis are presented to verify the validity and effectiveness of the proposed algorithm.

## 1. Introduction

Two-dimensional (2-D) direction-of-arrival (DOA) estimation of coherent signals has received much attention in many applications, such as radar, wireless communication and sonar in the multipath environment [1–5]. There are several high resolution techniques proposed to solve the rank deficiency of spatial covariance matrix caused by the presence of coherent signals. The conventional solution to this problem is the spatial smoothing method [6,7], which partitions the original array into a series of overlapping subarrays. Although it is efficient to decorrelate the incoming signals, peak searching of the spectrum in a 2-D space is required, which costs a large amount of computations. In order to reduce the computational complexity, an efficient method is performed by Hua [8]. This method, called the matrix enhancement and matrix pencil (MEMP) algorithm, exploits the structure inherent in an enhanced matrix from the original data. It estimates the azimuth and elevation separately in each dimension and combines them using a pairing method. However, the pairing result is not always correct when there are repeated parameters. Fortunately, a modified MEMP (MMEMP) method [9] is proposed to successfully solve the pairing problem.

In order to decorrelate the coherent signals thoroughly, recently, Han *et al.* [10] proposes an estimation of signal parameters via rotational invariance techniques (ESPRIT)-like algorithm for coherent DOA estimation. By reconstructing a Toeplitz matrix from the covariance matrix, this approach can decorrelate the impinging waves thoroughly. In [11], Chen extends it to the 2-D situation, namely the 2-D ESPRIT-like method, in conjunction with the MMEMP method, which outperforms the spatial smoothing method significantly in terms of the estimation accuracy. Although there is no peak searching existing in this algorithm, the computational burden is still heavy, due to the complex eigenvalue decomposition (EVD) and singular value decomposition (SVD) involved.

In this paper, we present a 2-D unitary ESPRIT-like (2-D UESPRIT-like) algorithm to reduce the computation complexity. Based on the block Hankel matrix obtained from [11], we preprocess it through a forward-backward average-like method convenient for unitary transformation. It can therefore transform the complex computations into real-valued ones and provide significant computational savings. The following DOA extractions are achieved simply by the one-dimensional (1-D) unitary ESPRIT [12], avoiding the computations of 2-D matrices. Simulation results will show that the real computations required for our new algorithm are much less than that of the 2-D ESPRIT-like method. It becomes especially obvious when the dimensionality of the Hankel matrix tends to be large. We also show that the variance of the estimates from our proposed method is close to the Cramer-Rao bound, and the resolution ability is superior to the others for the forward-backward average processing.

## 2. Background

### 2.1. Signal Model for URA

**Figure 1.**
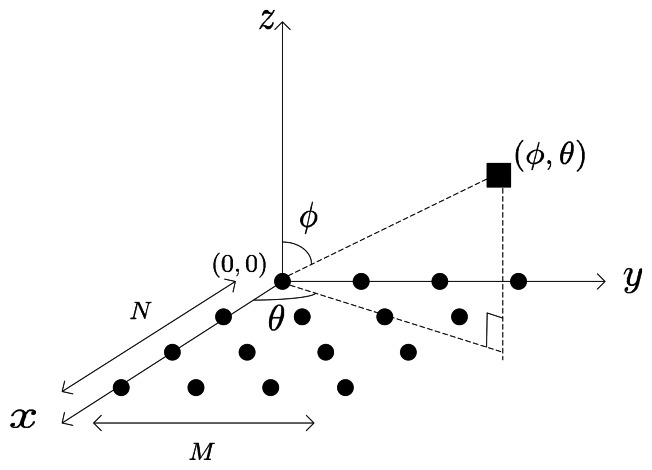
Sensor-source geometry configuration for 2-D direction-of-arrival (DOA) estimation.

Consider *K* narrowband, far-field and coherent radiating sources with wavelength λ impinging on a URA of *N* × *M* identical and omnidirectional sensors with interelement spacing, *d_x_* = *d_y_* = λ/2. Using analytic signal representation, the received signal at the (*n*, *m*)th sensor can be expressed by: [7]

(1) $$x_{n,m}(t)=\sum_{k=1}^{K}s_{k}(t)\beta_{k}^{-n}\gamma_{k}^{-m}+n_{n,m}(t)$$

where *s_k_*(*t*) is the complex envelope of the *k*th wavefront, (*β_k_*, *γ_k_*) = (e^jπ sin^*^ϕ^_k_*^cos^*^θ^_k_*, e^jπ sin^*^ϕ^_k_*^sin^*^θ^_k_*) and *ϕ_k_* and *θ_k_* are the elevation and azimuth angles of the *k*th source, respectively, and *n_n,m_*(*t*) is the additive spatially white noise with variance $\sigma_{n}^{2}$. Figure 1 shows the sensor-source geometry configuration in the 2-D scenario. For simplicity, we define:

(2) $$u_{k}=\pi \sin\phi_{k}\cos\theta_{k}\;\text{and}\;\upsilon_{k}=\pi \sin\phi_{k}\sin\theta_{k}$$

Therefore, (*β_k_*, *γ_k_*) can be expressed as (e^j^*^uk^*,e^j^*^υ^_k_*). Rewriting Equation (1) in vector notation, we get:

(3) x(t)=As(t)+n(t)

where ***x***(*t*) is the *NM* × 1 the data vector:

$$x(t)={\left[x_{0,0}(t),\ldots ,x_{0,M-1}(t),\ldots ,x_{N-1,0}(t)\ldots ,x_{N-1,M-1}(t)\right]}^{\text{T}}$$

***s***(*t*) is the *K* × 1 signal vector:

$$s(t)={\left[s_{1}(t),\ldots ,s_{K}(t)\right]}^{\text{T}}$$

***A*** is the *NM* × *K* steering vector matrix:

$$A=[a_{1},\ldots ,a_{K}]$$

with ***a****_k_* as the *NM* × 1 steering vector of the *k*th source:

$$a_{k}={\left[1,\ldots ,\beta_{k}^{-(N-1)}\right]}^{\text{T}}⊗{\left[1,\ldots ,\gamma_{k}^{-(M-1)}\right]}^{\text{T}}$$

and ***n***(*t*) is the *NM* × 1 noise vector:

$$n(t)={\left[n_{0,0}(t),\ldots ,n_{0,M-1}(t),\ldots ,n_{N-1,0}(t),\ldots ,n_{N-1,M-1}(t)\right]}^{\text{T}}$$

Here. we denote [·]^T^ as the transpose and ⊗ as the Kronecker product.

From Equation (3), it follows that the covariance matrix of the received signal is given by:

(4) $${\text{R}}_{x}=E[x(t)x{\left(t\right)}^{\text{H}}]=AE[s(t)s^{\text{H}}(t)]A^{\text{H}}+E[n(t)n^{\text{H}}(t)]$$

where *E* [·] denotes the expectation and (·)^H^ represents the complex conjugate transpose.

According to the derivation in [11], the element of ***R****_x_*, for example, the cross correlation of the signals received at the (*n*,*m*)th and (*p*,*q*)th sensor for *n*,*p* = 0,…, *N* − 1 and *m*, *q* = 0,…, *M* − 1 is given by:

(5) $$r(n,m;p,q)=\sum_{k_{2}=1}^{K}d_{k_{2},n,m}\beta_{k_{2}}^{p}\gamma_{k_{2}}^{q}+\sigma_{n}^{2}\delta_{n,p}\delta_{m,q}$$

where $d_{k_{2},n,m}=\sum_{k_{1}=1}^{K}E\left[s_{k_{1}}(t)s_{k_{2}}^{*}(t)\right]\beta_{k_{1}}^{-n}\gamma_{k_{1}}^{-m}$ with (·)* being the conjugate and $\delta_{n,p}=\{\begin{array}{cc} 1 & n=p\\ 0 & n\neq p \end{array}$.

### 2.2. Real-Valued Processing for 1-D ULA

As the real-valued processing with a uniform linear array (ULA) provides the important preliminary knowledge to our new algorithm, we give a quick review of the definition of the unitary matrix and the real-valued processing based on it, which have been widely used in certain kinds of unitary transformation algorithms ([12,13]*etc*). Suppose there are only *N* sensors located on the *x* axis; left in Figure 1. *N* is odd, and the center of the ULA is the reference. The DOA of the incoming signal is denoted by (*θ,ϕ* = π/2). Then, the *N* × 1 steering vector of ULA can be written as:

(6) $$\bar{a}(\theta )={\left[{\text{e}}^{-\text{j}\pi \frac{(N-1)\cos\theta }{2}},\ldots ,1,\ldots ,e^{\text{j}\pi \frac{(N-1)\cos\theta }{2}}\right]}^{\text{T}}$$

which is conjugate centro-symmetric. Such a property can be expressed mathematically as:

$$\prod_{N}\bar{a}(\theta )=\bar{a}*(\theta )$$

where **Π***_N_* is the *N* × *N* exchange matrix with ones on its antidiagonal and zeros elsewhere:

$$\prod_{N}={\left[\begin{array}{ccccc} 0 & 0 & \ldots & 0 & 1\\ 0 & 0 & \ldots & 1 & 0\\ \vdots & \vdots & \ddots & \vdots & \vdots \\ 0 & 1 & \ldots & 0 & 0\\ 1 & 0 & \ldots & 0 & 0 \end{array}\right]}_{N\times N.}$$

Define

(7) $$U_{N}=\frac{1}{\sqrt{2}}\left[\begin{array}{ccc} I_{\frac{N-1}{2}} & 0 & \text{j}I_{\frac{N-1}{2}}\\ 0^{\text{T}} & \sqrt{2} & 0^{\text{T}}\\ \prod_{\frac{N-1}{2}} & 0 & -\text{j}\prod_{\frac{N-1}{2}} \end{array}\right]$$

as the 1-D unitary matrix. By multiplying it, the elements in ***ā***)(*θ*) can be transformed into real quantities, such that:

(8) $$\begin{array}{ll} & {\text{U}}_{N}^{H}\bar{a}(\theta )\\ = & \sqrt{2}[\cos((N-1)\pi \cos(\theta )/2),\ldots ,\cos(\pi \cos\theta ),\\ & 1/\sqrt{2},\ldots ,-\sin((N-1)\pi \cos(\theta )/2),\\ & {\ldots ,-\sin(\pi \cos\theta )]}^{\text{T}} \end{array}$$

If *N* is even, a similar result can be obtained using:

(9) $$U_{N}=\frac{1}{\sqrt{2}}\left[\begin{array}{cc} I_{\frac{N}{2}} & \text{j}I_{\frac{N}{2}}\\ \prod_{\frac{N}{2}} & -\text{j}\prod_{\frac{N}{2}} \end{array}\right]$$

## 3. Proposed Algorithm

### 3.1. Signal Decorrelation

The proposed method is developed in the 2-D scenario, which has been introduced in Section 2.1. In order to resolve the rank deficiency problem caused by signal coherency, we first construct the following Hankel matrix from Equation (5) [11]:

$$R(n,m;p)=\left[\begin{array}{cccc} r(n,m;p,0) & r(n,m;p,1) & \ldots & r(n,m;p,M-Q)\\ r(n,m;p,1) & r(n,m;p,2) & \ldots & r(n,m;p,M-Q+1)\\ \vdots & \vdots & \ddots & \vdots \\ r(n,m;p,Q-1) & r(n,m;p,Q) & \ldots & r(n,m;p,M-1) \end{array}\right]$$

Then arranging a series of the above Hankel matrices into a block Hankel one, we have:

(10) $$R(n,m)=\left[\begin{array}{cccc} R(n,m;0) & R(n,m;1) & \ldots & R(n,m;N-P)\\ R(n,m;1) & R(n,m;2) & \ldots & R(n,m;N-P+1)\\ \vdots & \vdots & \ddots & \vdots \\ R(n,m;P-1) & R(n,m;P) & \vdots & R(n,m;N-1) \end{array}\right]$$

The analytic expression of ***R***(*n*, *m)* is given by [11]:

(11) $$\text{R}(n,m)=\text{BD}(n,m){\tilde{B}}^{\text{T}}$$

where ***B*** = [***b***_1_,…,***b****_K_*] with $b_{k}={\left[1,\beta_{k},\ldots ,\beta_{k}^{P-1}\right]}^{\text{T}}⊗{\left[1,\gamma_{k},\ldots ,\gamma_{k}^{Q-1}\right]}^{\text{T}}$, ***D***(*n*,*m*) = diag{*d*_1_,*_n_*,*_m_*,…,*d_K,n,m_*} and $\tilde{B}=[{\tilde{b}}_{1},\ldots ,{\tilde{b}}_{K}]$ with ${\tilde{b}}_{k}={\left[1,\beta_{k},\ldots ,\beta_{k}^{N-P}\right]}^{\text{T}}⊗{\left[1,\gamma_{k},\ldots ,\gamma_{k}^{M-Q}\right]}^{\text{T}}$. It has been proven that ***R***(*n*, *m*) is full-rank, provided the values of *P* and *Q* are selected properly. In this case, the rank of ***R***(*n*, *m*) equals the number of incoming signals. Therefore, lots of high resolution methods for 2-D DOA estimation of the uncorrelated or partly correlated signals can be used.

It should be noted that the following derivation will be performed under the assumption that there is no noise existing in the received data, which can be seen from Equation (11). Further study on the complex situation with spatially white noise will be carried out in Section 5 through several simulations.

### 3.2. Real- Valued Processing

Although we can apply the eigenstructure techniques to estimate 2-D DOA based on the full-rank ***R***(*n,m*), the computational burden is much heavier, because of the complex computations involved in it. In this note, we develop a 2-D unitary transformation method to reduce the complex computations to real ones.

If we premultiply and postmultiply ***R***(*n*, *m*) with unitary matrices directly, (*n*, *m*) cannot be transformed into real-valued, because the matrix ***D***(*n*, *m*) is complex. Therefore, we need to construct a new matrix associated with ***R***(*n*, *m*) before unitary transformation to guarantee this property. Let us define:

(12) $$\text{Y}=[\text{R}(n,m)\prod_{PQ}\text{R}*(n,m)]$$

Then we have:

(13) $$\begin{array}{cc} R_{Y} & =YY^{\text{H}}\\ & =R(n,m)R^{\text{H}}(n,m)+\prod_{PQ}(R(n,m)R^{\text{H}}(n,m))*\prod_{PQ}\\ & =BD(n,m){\tilde{\text{B}}}^{\text{T}}\tilde{\text{B}}*{\text{D}}^{\text{H}}(n,m)B^{\text{H}}+\prod_{PQ}B*D*(n,m){\tilde{B}}^{\text{H}}\tilde{B}D^{\text{T}}(n,m)B^{\text{T}}\prod_{PQ} \end{array}$$

With the property of the Kronecker product *α*_1_*α*_2_(***B***_1_⊗***B***_2_) = *α*_1_***B***_1_⊗*α*_2_***B***_2_, where *α*_1_ and *α*_2_ represent any constant and ***B***_1_ and ***B***_2_ represent matrices, ***B*** can thus be converted into:

(14) B=B'C

Furthermore, we have

(15) $$\prod_{PQ}B*=B\prime C*$$

where ***C*** = diag{e^j [(^*^P^*^−1)^*^u^*^1+(^*^Q^*^−1)^*^υ^*^1]/2^,…, e^j [(^*^P^*^−1)^*^uK^*^+(^*^Q^*^−1)^*^υK^*^]/2^}, $B\prime =[b_{1}^{\prime },\ldots ,b_{K}^{\prime }]$ with $b_{k}^{\prime }=\bar{b}(u_{k})⊗\bar{b}(\upsilon_{k})$, ***b̄***(*u_k_*) = [e^−j(^*^P^*^−1)^*^uk^*^/2^,…,e^j(^*^P^*^−1)^*^uk^*^/2^]^T^ and ***b̄***(*υ_k_*) = [e^−j(^*^Q^*^−1)^*^υk^*^/2^,…,e^j(^*^Q^*^−1)^*^υk^*^/2^]^T^. ***b̄***(*u_k_*) and ***b̄***(*υ_k_*) are both conjugate centro-symmetric, the same as the steering vector given by Equation (6).

Substituting Equations (14) and (15) into Equation (13)***R****_Y_* can be rewritten as:

(16) $$R_{Y}=B\prime (F+F*)B\prime^{\text{H}}$$

where ***F*** + F* is real, with ***F*** = ***CD***(*n*,*m*)***B̃***^T^B̃*D^H^(*n*,*m*)***C***^H^. Compared with Equation (4)***B****′* in Equation (16) can be viewed as a new array response matrix and ***F*** + F* as the equivalent covariance matrix of the incoming signals. Notice that the achievement of ***R****_Y_* is consistent with the forward-backward average processing [14,15]. Compared with ***R***(*n*,*m*), ***R****_Y_* decorrelates the coherent signals more thoroughly and, therefore, can provide higher estimation accuracy.

Since ***b̄***(*u_k_*) and ***b̄***(*υ_k_*) in ***B****′* have the similar form as Equation (6), we can make use of the 1-D real-valued processing-like Equation (8) to perform the 2-D unitary transformation. Define the 2-D unitary matrix as:

$${\text{U}}_{P,Q}={\text{U}}_{P}⊗{\text{U}}_{Q}$$

where ***U****_P_* and ***U****_Q_* use the definition of Equation (7) or (9). Premultiplying and postmultiplying ***R****_Y_* by ***U****_P,Q_*, yields:

(17) $$\varphi =U_{P,Q}^{\text{H}}R_{Y}U_{P,Q}$$

According to the useful property:

(18) $$A_{1}A_{2}⊗B_{1}B_{2}=(A_{1}⊗B_{1})(A_{2}⊗B_{2})$$

*φ* becomes:

$$\begin{array}{cc} \varphi & ={\left(U_{P}⊗U_{Q}\right)}^{\text{H}}B\prime (F+F*)B\prime^{\text{H}}(U_{P}⊗U_{Q})\\ & =B_{u}^{H}(F+F*)B_{u} \end{array}$$

with ${\text{B}}_{u}^{\text{H}}=[({\text{U}}_{P}^{\text{H}}\bar{b}(u_{1}))⊗(U_{Q}^{\text{H}}\bar{b}(\upsilon_{1})),\ldots ,(U_{P}^{\text{H}}\bar{b}(u_{K}))⊗(U_{Q}^{\text{H}}\bar{b}(\upsilon_{K}))]$. As the vector in each column of $B_{u}^{\text{H}}$, for example $U_{P}^{\text{H}}\bar{b}(u_{1})$, satisfies Equation (8), the matrix ***B****_u_* is real. Combined with the real-valued ***F*** + F*, we can easily deduced that *ϕ* is real.

### 3.3. Extracting β_k_ and γ_k_

To avoid peak searching, to retain the 2-D DOA estimation real-valued and reduce the computational complexity, we develop a simple implement of the 2-D unitary ESPRIT based on the 1-D solution [12].

Let eigenvectors associated with the *K* largest eigenvalues of ***R****_Y_* be denoted by ***E****_sRY_* ∈ **C***^PQ^*^×^*^K^*. Since ***E****_sRY_* and ***B****′* both span the signal subspace of ***R****_Y_*, as Equation (16) has shown, there exists a unique, nonsingular matrix, ***T*** ∈ **C***^K^*^×^*^K^*, such that ***E****_sRY_* = ***B****′****T***. Define ***E****_s_*_1_ = ***J***_1_***E****_sRY_* and ***E****_s_*_2_ = ***J***_2_***E****_sRY_* as the first and last, (*P* − 1)*Q*, rows of matrix, ***E****_sRY_*, respectively, with ***J***_1_ = [***I***_(_*_P_*_−1)_*_Q_*, **0**_(_*_P_*_−1)_*_Q_*_×_*_Q_*] and ***J***_2_ = [**0**_(_*_P_*_−1)Q×Q_, ***I***_(_*_P_*_−1)_*_Q_*]. Then, replacing ***E****_sRY_* by ***B****′****T***, we have:

(19) $$E_{s1}=J_{1}B\prime T,E_{s2}=J_{2}B\prime T$$

Observing the structure of ***B****′*, we can find that:

(20) $${\text{J}}_{2}\text{B}\prime ={\text{J}}_{1}\text{B}\prime \Omega_{u}$$

with Ω*_u_* = diag{e^ju1^,…, *e*^j^*^uK^*}. Combined with Equations (19) and (20), we can write the relationship between ***E****_s_*_1_ and ***E****_s_*_2_ as:

(21) $${\text{E}}_{s2}={\text{E}}_{s1}\Psi_{u},\text{where}\Psi_{u}=T^{-1}\Omega_{u}T$$

It is clear that Ψ*_u_* is the total least squares (TLS) solution of Equation (21). The eigenvalues of Ψ*_u_* are equal to the diagonal elements of Ω*_u_*. Therefore, *u_k_* corresponding to *β_k_* can be extracted by an EVD of Ψ*_u_*. In [12], the author has proved that the TLS problem can be solved through a SVD of [***E****_s_*_1_, ***E****_s_*_2_]. Here, we define:

(22) $$U_{(P-1),Q}=U_{P-1}⊗U_{Q}$$

By reconstructing the matrix, [***E****_s_*_1_, ***E****_s_*_2_], and performing the unitary transformation, the TLS problem can be solved by computing the SVD of the real matrix:

(23) $${\text{T}}_{1}={\text{U}}_{(P-1),Q}^{\text{H}}[{\text{E}}_{s1},E_{s2}\prod_{K}]U_{2K}$$

with ***U***_2_*_K_* defined by Equation (9). Note the eigenvectors are associated with the *K* largest eigenvalues of matrix, *ϕ*, as ***E****_sϕ_*. From Equation (17), we have:

(24) $${\text{E}}_{s\varphi }={\text{U}}_{P,Q}^{\text{H}}{\text{E}}_{sR_{Y}}$$

Substituting Equations (22) and (24) into Equation (23) and rearranging it, yields:

(25) $$\begin{array}{cc} T_{1} & =U_{(P-1),Q}^{\text{H}}[J_{1}U_{P,Q}{\text{E}}_{s\varphi },J_{2}U_{P,Q}{\text{E}}_{s\varphi }]\frac{1}{\sqrt{2}}\left[\begin{array}{cc} I_{\text{K}} & \text{j}I_{K}\\ \prod_{K} & -\text{j}\prod_{K} \end{array}\right]\\ & =\frac{1}{\sqrt{2}}[K_{1}E_{s\varphi },K_{2}E_{s\varphi }] \end{array}$$

where $K_{1}=U_{(P-1),Q}^{\text{H}}(J_{1}+J_{2})U_{P,Q}$ and $K_{2}=\text{j}U_{(P-1),Q}^{\text{H}}(J_{1}-J_{2})U_{P,Q}$Since the computations of ***K***_1_, ***K***_2_ require a large amount of multiplies, we intend to simplify their expressions with lower dimensions. Let

$$J_{1}^{\prime }=[I_{P-1},0_{(P-1)\times 1}]\;\text{and}\;J_{2}^{\prime }=[0_{(P-1)\times 1},I_{P-1}]$$

which are selection matrices used in the 1-D case [12]. It follows that $J_{1}=J_{1}^{\prime }⊗I_{Q}$ and $J_{2}=J_{2}^{\prime }⊗I_{Q}$. Using the property Equation (18)***K***_1_ can be simply determined by:

(26) $$\begin{array}{cc} K_{1} & =(U_{P-1}^{H}⊗U_{Q}^{H})(J_{1}^{\prime }⊗I_{Q}+J_{2}^{\prime }⊗I_{Q})(U_{P}⊗U_{Q})\\ & =K_{1}^{\prime }⊗I_{Q} \end{array}$$

where $K_{1}^{\prime }=U_{P-1}^{H}(J_{1}^{\prime }+J_{2}^{\prime })U_{P}$. As [12] stated, the matrix $K_{1}^{\prime }$ is real. Similarly, we have:

(27) $$K_{2}=K_{2}^{\prime }⊗I_{Q}$$

with $K_{2}^{\prime }=\text{j}U_{P-1}^{H}(J_{1}^{\prime }-J_{2}^{\prime })U_{P}$. Therefore, the computations of ***T***_1_ in Equation (25) can be greatly simplified by using Equations (26) and (27).

Denote the right singular vector matrix of ***T***_1_ by ***W****_u_* and partition it into submatrices:

$$W_{u}=\left[\begin{array}{ll} w_{u11} & w_{u12}\\ w_{u21} & w_{u22} \end{array}\right]\in R^{2K\times 2K}$$

Finally, we get the real-valued TLS solution corresponding to Ψ*_u_* as:

(28) $${ϒ}_{u}=-w_{u12}w_{u22}^{-1}$$

To extract the parameter *υ_k_*, we define different *P*(*Q* − 1) rows of ***E****_sRY_* as ***E****_s_*_3_ = ***J***_3_***E****_sRY_* and ***E****_s_*_4_ = ***J***_4_***E****s_RY_*, in which ${\text{J}}_{3}={\text{I}}_{P}⊗J_{3}^{\prime }$ and $J_{4}=I_{P}⊗J_{4}^{\prime }$ with:

$$J_{3}^{\prime }=[I_{Q-1},0_{(Q-1)\times 1}],J_{4}^{\prime }=[0_{(Q-1)\times 1}I_{Q-1}]$$

Similar to the derivation of Equation (21), we have:

$$E_{s4}=E_{s3}\Psi_{\upsilon }\text{with}\Psi_{\upsilon }=T^{-1}\Omega_{\upsilon }T$$

where Ω*_υ_* = diag{e^j^*^υ^*^1^, …,e^j^*^υK^*} and ***T*** are the nonsigular matrix in Equation (19). Ψ*_υ_* is the TLS solution, which can be obtained by the SVD of real matrix:

(29) $$T_{2}=\frac{1}{\sqrt{2}}[K_{3}E_{s\varphi },K_{4}E_{s\varphi }]$$

where $K_{3}=I_{P}⊗K_{3}^{\prime },K_{4}=I_{P}⊗K_{4}^{\prime }$ with:

$$K_{3}^{\prime }=U_{Q-1}^{H}(J_{3}^{\prime }+J_{4}^{\prime })U_{Q},K_{4}^{\prime }=\text{j}U_{Q-1}^{H}(J_{3}^{\prime }+J_{4}^{\prime })U_{Q}$$

Partitioning the right singular vector matrix of the real *P*(*Q* − 1) × 2*K* matrix ***T***_2_ into a block one, we obtain:

$$W_{\upsilon }=\left[\begin{array}{ll} w_{\upsilon 11} & w_{\upsilon 12}\\ w_{\upsilon 21} & w_{\upsilon 22} \end{array}\right]\in R^{2K\times 2K}$$

Then, it follows that the real-valued TLS solution associated with Ω*_υ_* can be computed by:

(30) $${ϒ}_{\upsilon }=-w_{\upsilon 12}w_{\upsilon 22}^{-1}$$

Employing the existing automatic pairing method [16,17], the estimates, *υ_k_* and *υ_k_*, can be achieved by computing the EVD of the “complexified” matrix **ϒ***_u_* + j**ϒ***_υ_*. Denote the real and the imaginary parts of the *K* eigenvalues as ${\left\{r_{uk}\right\}}_{k=1}^{K}$ and ${\left\{r_{\upsilon k}\right\}}_{k=1}^{K}$. The estimation of *u_k_* and *υ_k_* will be:

(31) $$u_{k}=2\arctan(r_{uk}),\upsilon_{k}=2\arctan(r_{\upsilon k})$$

From Equation (2), we can get the final result:

(32) $$\theta_{k}=\arctan(\upsilon_{k}/u_{k}),\phi_{k}=\arcsin\sqrt{u_{k}^{2}+\upsilon_{k}^{2}}/\pi$$

### 3.4. Summary of the Algorithm

The steps of the proposed method are described as follows:

Step 1. Obtain ***X*** = [*x*(*t*_1_),…, *x*(*t_L_*)] with *x*(*t_k_*) as the snapshot at time, *t_k_*. Then, compute the covariance matrix approximately by ***R****_x_* = ***XX****^H^/L* with *L* as the snapshot number.
Step 2. Construct the block Hankel matrix ***R***(*n*, *m*) by Equation (10) to decorrelate the coherency of signals and obtain ***R****_Y_* through Equations (12) and (13) to facilitate the following unitary transformation. Then, compute the real-valued matrix $\varphi =U_{P,Q}^{H}R_{Y}U_{P,Q}$.
Step 3. Compute ***E****_sϕ_* as the *K* dominant eigenvectors of *ϕ* and calculate ***T***_1_, ***T***_2_ through Equations (25) and (29). Conduct the SVD of ***T***_1_, ***T***_2_ to obtain the right singular vector matrices, ***W****_u_*, ***W****_υ_*, and get ϒ_u_, ϒ*_υ_* from Equations (28) and (30), respectively.
Step 4. Conduct EVD of the complex-valued matrix, **ϒ**_u_ + j**ϒ***_υ_*. Extract *u_k_* and *υ_k_* from the eigenvalues by Equation (31). At last, estimate *θ_k_* and *ϕk* using Equation (32).

## 4. Computational Order Analysis

In the following, we will first derive an estimate of the order of real multiplications involved in each step. Then, we will compare the computational order of our new method, namely the 2-D UESPRIT-like method, against that of the 2-D ESPRIT-like method.

### 4.1. Computational Order of Step 1

Here, we calculate ***R****_x_* by ***XX***^H^*/L* with ***X*** ∈ *C^MN^*^×^*^L^*. The direct computation requires $4\times \frac{1}{2}MN(MN+1)L$ real multiplications.

### 4.2. Computational Order of Step 2

From Equation (13), we can see that to obtain ***R****_Y_*, only the computation of ***R****_y_* = ***R***(*n*, *m*)***R***^H^ (*n*, *m*) is needed. It is because $\prod_{PQ}R_{y}^{*}\prod_{PQ}$ can be achieved by rearranging the elements of ***R****y* simply, without any multiplication. According to the computational analysis in [8], the minimum number of real multiplications required to compute ***R****_y_* is:

4×[2PQ(N−P/2)(M−Q/2)]

provided *P* ≫ 1,*Q Gt;* 1,*N* − *P Gt;* 1,*M* − *Q Gt;* 1.

In this step, we also need to calculate *ϕ*. Due to the special structure of unitary matrices, *U_P_* and *U_Q_*, the computation of ***U****_P,Q_* = ***U****_P_* ⊗ ***U****_Q_* only contains that of the product of j and *U_Q_*. Therefore, the order of computing ***U****_P,Q_* is 2*Q*. For the same reason, the real multiplications involved in $\varphi =U_{P,Q}^{H}R_{Y}U_{P,Q}$ is:

$$[PQ\times \frac{Q}{2}\times P+(PQ+4)\times \frac{Q}{2}\times P]\times 2=2{\left(PQ\right)}^{2}+4PQ$$

### 4.3. Computational Order of Step 3

As *φ* is a real-valued matrix, the number of multiplications required (based on the symmetric QR algorithm [18]) for its EVD is:

(33) $$5{\left(PQ\right)}^{3}$$

when *PQ* ≫ 1.

The following real multiplications involved in the **ϒ***_u_* and **ϒ***_υ_* achievement is listed in Table 1. Denote the total number involved in it as *C*_1_. The computational order of SVD is obtained by the Chan SVD [18].

### 4.4. Computational Order of Step 4

In this step, only the EVD of complex-valued matrix, **ϒ***_u_* + j**ϒ***_υ_*, is considered. It requires 4 × 5*L*^3^ real multiplications when *L*≫1.

### 4.5. Comparison to the 2-D ESPRIT-Like Method

According to the above analysis, the order of computations needed by the 2-D UESPRIT-like method is:

(34) $$\begin{array}{ll} & 2MN(MN+1)L+4[2PQ(N-P/2)(M-Q/2)]\\ & +8{\left(PQ\right)}^{2}+5{\left(PQ\right)}^{3}+20L^{3}+C_{1}\\ \approx & 4[2PQ(N-P/2)(M-Q/2)]+5{\left(PQ\right)}^{3} \end{array}$$

as long as*P* > *L* ≫ 1,*Q* > *L* ≫ 1,*N* ≫ 1,*M* ≫ 1. It can be seen that the computation of ***R****_y_* and the EVD of *ϕ* occupy the major part.

In contrast, we present the computations needed in the 2-D ESPRIT-like method [11], which uses the same decorrelation processing, but a different MMEMP method behind it. As stated in [8], the multiplications required in each step are listed in Table 2. *C*_2_ = 3*PQL*^2^ + 7*L*^3^, the number of complex multiplications used in MMEMP, is obtained under the assumption that there are no repeated *β_k_*. From Table 2, the real computations of the 2-D ESPRIT-like method can be written as:

(35) $$\begin{array}{ll} & 2MN(MN+1)L+4[2PQ(N-P/2)(M-Q/2)\\ & +5{\left(PQ\right)}^{3}]+3PQL^{2}+7L^{3}\\ \approx & 4[2PQ(N-P/2)(M-Q/2)+5{\left(PQ\right)}^{3}] \end{array}$$

According to Equations (34) and (35), the computational saving of our method is about 15(*PQ*)^3^, caused by the unitary transformation. In [8], the author recommended the scope of *P*, *Q* given below:

L+1≤P≤(N+1)/2,L+1≤Q≤(M+1)/2

in which the estimation accuracy and computations become both higher as the values increase. For comparison, we estimate the most multiplications cost in computing ***R***(*n*, *m*) by choosing *P* = (*N* + 1)/2 and *Q* = (*M* + 1)/2. In this case, the first part of Equations (34) and (35) becomes:

(36) $$4\times [2PQ(N-P/2)(M-Q/2)]\approx 18{\left(PQ\right)}^{2}$$

In the condition of *P* > *L* ≫ 1 and *Q* > *L* ≫ 1, Equation (33) is far more than Equation (36), which indicates that the computational saving of our algorithm is considerable.

**Figure 2.**
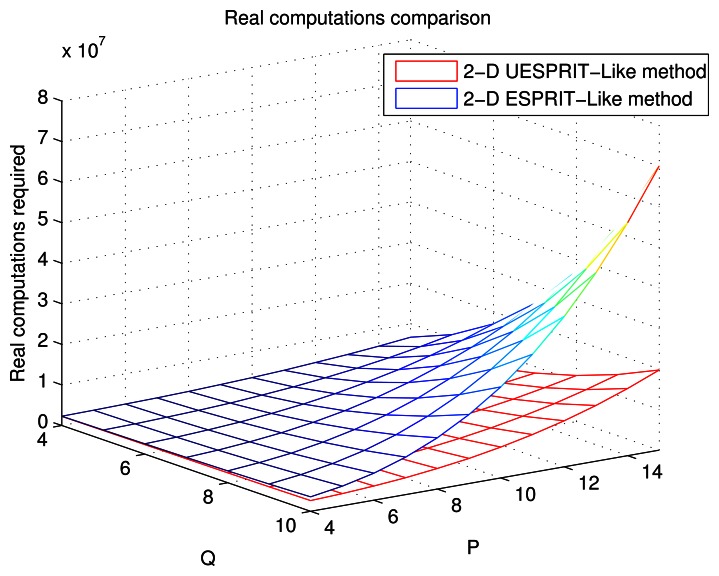
Real computations needed to estimate the 2-D DOA as a function of P and Q.

Figure 2 is plotted to compare the proposed method with the 2-D ESPRIT-like method in the aspect of real computations required as a function of P and Q. The multiplications cost in each method are computed by the sum of the corresponding column in Table 2. The snapshot number is *L* = 3, and the size of the URA is *N* × *M* = 30 × 20. *P* and *Q* change in the scope of [*L* + 1, (*N* + 1)/2] and [*L* + 1, (*M* + 1)/2]. The figure shows that as *P* and *Q* increase, the complexities of estimating the 2-D DOA with two different algorithms increase, as well. The computations needed for our new method is much less than that of the 2-D ESPRIT-like method when *P* and *Q* go towards the upper bound. Therefore, we conclude that the proposed scheme can obtain dramatic computational savings in estimating the elevation and azimuth.

## 5. Simulation Results

In this section, we present simulation results that compare the proposed method with several other 2-D DOA estimations in the presence of a zero mean Gaussian white noise. Except the developed scheme, DOA extractions and pairings in other methods are all performed using MMEMP [9].

Suppose *K* narrowband equipowered signals are incident on a 11 × 9 URA (*i.e.*, *N* = 11, *M* = 9) with interelement spacing *d_x_* = *d_y_* = λ/2. The correlation factor between the first signal and the others is denoted as *γ_s_*. When *γ_s_* equals 1, they are coherent and completely uncorrelated when *γ_s_* = 0. We generate correlated signals via:

$$\begin{array}{c} s_{1}(t)=\sqrt{\text{SNR}}\times r_{1}(t)\\ s_{k}(t)=(s_{1}(t)\sqrt{\gamma_{s}}+r_{k}(t)\sqrt{\left(1-\gamma_{s}\right)})\times \sqrt{\text{SNR}}\text{for}k=2,3\ldots ,K \end{array}$$

where *s_k_*(*t*) is the amplitude of the *k*th signal at time, *t*, SNR denotes the signal to noise ratio and *r_k_*(*t*) is the output of the *k*th random number generator at time, *t*. In the range of Equation (36), we select *P* = 5 and *Q* = 4.

**Figure 3.**
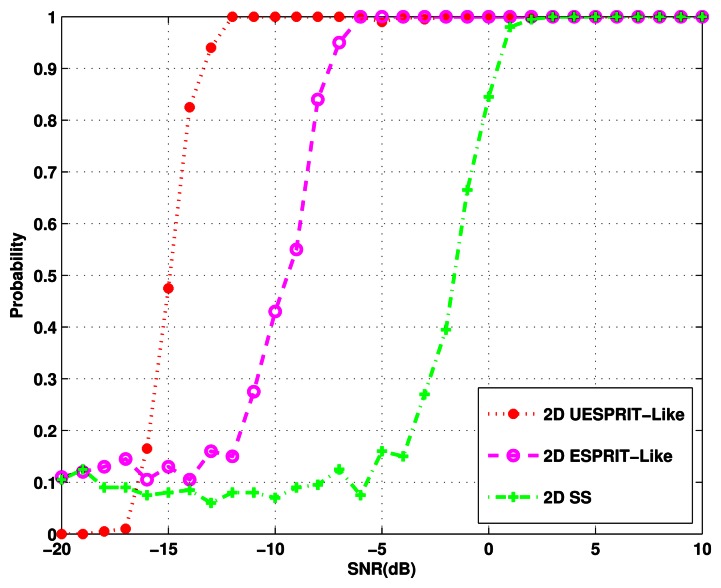
Probability of the DOA identification *versus* input signal to noise ratio (SNR).

First, we consider three coherent signals from (*ϕ, θ*) = (10°, 0°), (5°,−5°), (−5°, 7°) with SNR varying from −20 dB to 10 dB. 1,000 Monte Carlo trials are run for each SNR. Five hundred snapshots are taken to form the estimate of the covariance matrix of the array outputs. Figure 3 shows the probability of identifying all the directions correctly *versus* SNR. The result illustrates that the performance of both the proposed algorithm and 2-D ESPRIT-like method are better than that of 2-D spatial smoothing (2-D SS). This is because the first two algorithms can eliminate the coherency of signals completely by reconstructing the equivalent covariance matrix ***R***(*n*, *m*) by  Equation 10, while the 2-D SS method can only provide the alleviation of the rank deficiency to some extent. The graph also shows that the new method has some improvement over the 2-D ESPRIT-like method, due to the fact that the achievement of ***R****_Y_* in our method is similar to the forward-backward spatial smoothing process [14], which can further enhance the ability of decorrelation.

**Figure 4.**
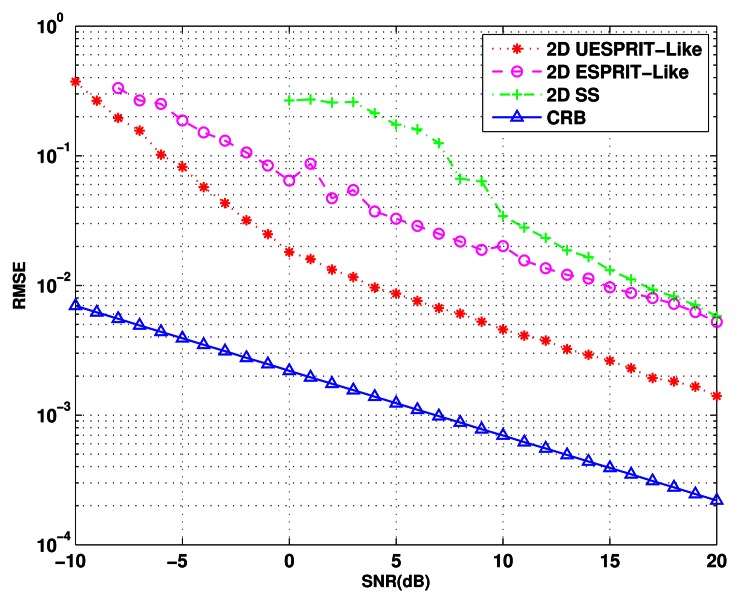
Root mean square error (RMSE) of the DOA estimates *versus* input SNR.

Second, we consider the same scenario as in the first one. Define the root mean square error (RMSE) of the DOA estimates as:

$$\text{RMSE}=\sqrt{\frac{1}{1000K}\sum_{k=1}^{K}\sum_{n=1}^{1000}\left[{\left({\hat{\theta }}_{k}^{\left(n\right)}-\theta_{k}\right)}^{2}+{\left({\hat{\phi }}_{k}^{\left(n\right)}-\phi_{k}\right)}^{2}\right]}$$

where ${\hat{\theta }}_{k}^{n}$ and ${\hat{\phi }}_{k}^{n}$ are the estimates of *θ_k_* and *ϕ_k_* for the *n*th trial,respectively. *K* is the number of signals. The comparison of Cramer-Rao lower bound (CRB), computed according to formulas provided in [19] and the RMSE of DOA estimates of 2-D UESPRIT-like method, 2-D ESPRIT-like method and 2-D SS are plotted in Figure 4. The SNR changes from −10 dB to 20 dB. Simulation result shows that the estimation errors of all the methods decrease obviously as the SNR increase. Moreover, our proposed method is observed to have a superior performance over the others and to be close to the CRB most. When the SNR is lower than 0 dB, the 2-D SS method fails to distinguish the two closely located sources, while our algorithm can still accomplish it very well. The same phenomenon appears for the 2-D ESPRIT-like method when the SNR is lower than −7 dB.

**Figure 5.**
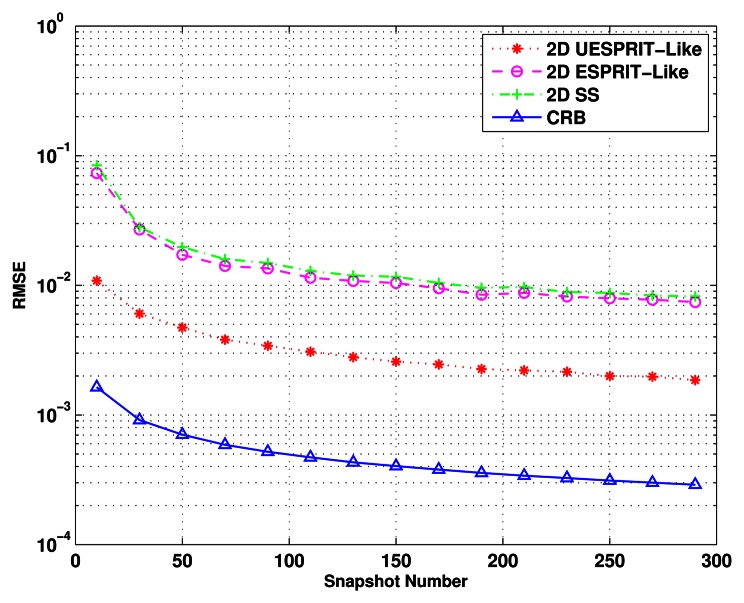
RMSE of the DOA estimates *versus* snapshot number.

The third simulation considers the same signals as the above two experiments with SNR = 20 dB. The snapshot number, *L*, changes from 10 to 300. The RMSE of the 2-D estimates as a function of the snapshot number and the CRB are plotted in Figure 5. As expected, the 2-D UESPRIT-like method is shown to have dramatically high accuracy over the other two and can achieve the closest performance to CRB.

**Figure 6.**
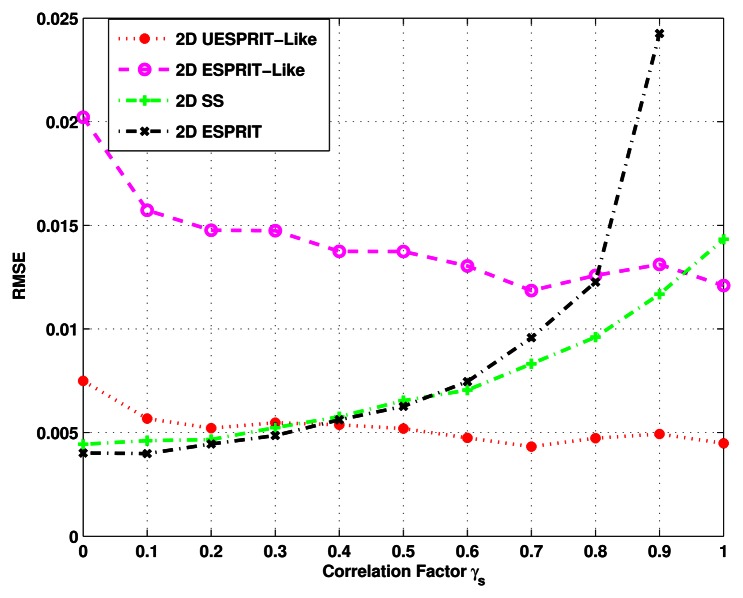
RMSE of the DOA estimates *versus* correlation factor.

In the last simulation, we investigate the ability of four algorithms to decorrelate coherent signals. Assume there are two narrowband signals with (*ϕ, θ*) = (10°, 0°), (5°, −5°) and SNR = 5dB. Their correlation factor varies in the range (0,1). For each value of *γ_s_*, 1,000 independent estimates are carried out to examine the RMSE of DOA estimates. First, we compare the 2-D ESPRIT method with the other three decorrelation algorithms. As Figure 6 has shown, when the signals are uncorrelated or partly correlated with low correlation factor, the conventional 2-D ESPRIT is the best choice for DOA estimation. The reason is the use of decorrelation in the other methods results in a small effective aperture, which can reduce the resolution significantly. However, as *γ_s_* increases, the performance of the 2-D ESPRIT degrades slowly. Until *γ_s_* = 0.9, it totally fails to identify DOA, because of the serious rank loss of ***R****_x_*. Besides, Figure 6 also demonstrates that the 2-D SS method provides a much better performance than the 2-D ESPRIT-like method, as well as our proposed method, when *r_s_* ≤ 0.32. This is due to the fact that in such a low correlation, it is sufficient for 2-D SS to decorrelate signals, and it can restrain noise to some extent. When the signals become nearly coherent, that is *γ_s_* ≥ 0.95, the superiority of our proposed scheme and the 2-D ESPRIT-like method appears to be remarkable for the excellent decorrelation ability, while in terms of the veracity of DOA estimates, it is obvious that the new method outperforms 2-D ESPRIT-like method all the time.

## 6. Conclusions

An application of unitary transformation to 2-D DOA estimation of coherent sources has been proposed in this paper. The decorrelation is performed based on the existing 2-D ESPRIT-like method. While the computational load is significantly reduced by transforming the complex matrices into real-valued ones and conducting the EVD with 1-D matrices, the 2-D UESPRIT-like method can also provide better performance in DOA estimation by preprocessing the block Hankel matrix using the forward-backward averaging-like method. Computational analysis and simulation results have shown the significant reduction of computations and the dramatic low RMSE in DOA estimations. A less restrictive requirement for the array geometry is also provided to generalize the application of this method.

It is worth mentioning that our proposed algorithm is fit for dealing with the estimation of highly correlated signals. In the situation where all the signals are uncorrelated or partly correlated, the method given in this paper will suffer degradation to some extent.

It is also interesting to notice that our new algorithm is still practically useful in the presence of mutual coupling, though such an effect was not considered in this paper. For 2-D DOA estimation in URA, if the mutual coupling matrix (MCM) is known, the coupling effect can be easily eliminated by premultiplying the inverse MCM with the original data. If the MCM is unknown, we can also use the method provided in [20] to completely alleviate the mutual coupling by setting the sensors on the boundary of the URA as auxiliary sensors, provided that the MCM satisfies the block banded symmetric Toeplitz assumption. The output of the middle URA is, therefore, an ideal model without a coupling effect. Moreover, the 1-D version of our proposed method, namely 1-D UESPRIT [21], can be extended to the azimuth estimation with the Uniform Circular Array (UCA) in the presence of mutual coupling. In this case, the elevation is assumed to be known. The mutual coupling can be directly compensated for if the coupling effect is not so serious and the MCM is known [22]. Then, the array manifold of the UCA is projected by a coupling matrix onto that of an ideal UCA, where the azimuth estimation is the same as that of ULA, and the 1-D UESPRIT method can be directly conducted. However, it cannot be applied to the method proposed in [23] when the number of antenna elements in the UCA is large enough, as the *H* matrix used to incorporate all the relevant phase modes into the principal term destroys the Vandermode structure of the beamspace steering vector. Besides, the new method may not be applied as well in the more realistic situation of 2-D DOA estimation with UCA, as the UCA ESPRIT and UCA-ESPRIT-like involved in the algorithms [19,24] are different from the 2-D ESPRIT of URA [17]. Moreover, the elevation-dependence of MCM prevents the application of our approach to the method in [25]. Future work may focus on the real-valued processing of UCA ESPRIT, utilizing the special structure of unitary matrix.

## Figures and Tables

**Table 1.** Real multiplications involved in the computations of **ϒ***_u_* and **ϒ***_υ_*.

	**Real multiplications**	**conditions**
${\text{K}}_{1}^{\prime },{\text{K}}_{2}^{\prime }$	2 [(*P* − 1)^2^*P* + *P*^2^(*P* − 1)]	/
***K***_1_,***K***_2_	2*PQ*(*P* − 1)	/
***K***_1_***E****_sϕ_*, ***K***_2_***E****_sϕ_*	2*PQ*^2^(*P* − 1)*L*	/
SVD of ***T***_1_	(2*L*)^2^(*P* − 1)*Q*+ 17(2*L*)^3^/3	*PQ* > (*P* −1)*Q*
**ϒ** *_u_*	*L* ^3^	/

$K_{3}^{\prime },K_{4}^{\prime }$	2 [(*Q* − 1)^2^*Q* + *Q^2^*(*Q* − 1)]	/
***K***_3_,***K***_4_	2*PQ*(*Q* − 1)	/
***K***_3_***E****_sϕ_*, ***K***_4_***E****_sϕ_*	2*P*^2^*Q*(*Q* − 1)*L*	/
SVD of ***T***_2_	(2*L*)^2^*P*(*Q* − 1)+ 17(2*L*)^3^/3	*PQ* > *P*(*Q* − 1)
**ϒ** *_υ_*	*L* ^3^	/

**Table 2.** Real multiplications required for the 2-D UESPRIT-like and 2-D ESPRIT-like method.

	**2D UESPRIT-Like Method**	**2D ESPRIT-Like Method**
***R*** *_x_*	2*M N*(*M N* +1)*L*	2*M N*(*M N* +1)*L*
***R*** *_y_*	4 × [2*PQ*(*N* − *P*/2)(*M* − *Q*/2)]	4 × [2*PQ*(*N* − *P*/2)(*M* − *Q*/2)]
*φ*	2(*PQ*)^2^ + 4*PQ*	/
EVD	of *φ*: 5(*PQ*)^3^	of ***R****_y_*: 20(*PQ*)^3^
The rest of the operations	2D Unitary ESPRIT:*C*_1_ + 20*L*^3^	MMEMP: *C*_2_
